# The impact of weather anomalies on violence in the coastal mid-latitudes: a cross-national comparison

**DOI:** 10.1007/s00484-024-02762-x

**Published:** 2024-09-06

**Authors:** Gregory Breetzke, Jonathan Corcoran

**Affiliations:** 1 Department of Geography Geoinformatics and Meteorology, University of Pretoria, Pretoria, South Africa; 2 School of the Environment, Faculty of Science, University of Queensland, Brisbane, Australia

**Keywords:** Violent crime, Temperature, Rainfall, Khayelitsha, Ipswich

## Abstract

Cross-national studies examining the relationship between weather and crime are rare. Reasons are manifold but include the differences in countries due to geographical, climatic, and seasonal variations. In contrast in this study we examine the causal impact of temperature and rainfall anomalies on violent crime in locations located in two comparable geographic zones: Khayelitsha (in South Africa) and Ipswich (in Australia). We use ANOVA and Tukey’s tests to identify statistical meaningful differences (if any) in the impact of these weather anomalies on crime alongside the use of visualisations capturing the anomalous weather-violence relationship in these two contexts. Results show some similarities but also notable differences between locations which we attribute to their inherent socio-demographic differences which we expand upon. We conclude by highlighting the benefits of cross-national crime research, and motivate for its increased application in future research of this nature.

**Supplementary Information:**

The online version contains supplementary material available at 10.1007/s00484-024-02762-x.

## Introduction

One of the least disputed axioms in criminology is that weather impacts crime. Research across a broad swathe of countries has most often found significant positive associations between crime and a range of meteorological parameters including temperature (Xiaofeng et al. 2017; Stevens et al. 2019), rainfall (Hsiang et al. 2013; Shen et al. 2020), wind speed (Hart et al. 2022), and humidity (Schutte and Breetzke 2018), with a few exceptions (see Pittman and Handy 1964; Yan 2004). The sociological explanation, borne largely out of routine activities theory (Cohen and Felson 1979), is that weather affects individuals’ behavioural patterns which places them at an increased (or decreased) risk of victimisation. The RA theory argues that individuals generally follow strict temporal routines (daily, weekly and monthly), which affects opportunities for crime (Brunsdon et al. 2009). While some of these activities are compulsory with a fixed duration (such as school or work), others are more flexible (such as socialising) leaving individuals with a greater amount of choice as to when or where they will occur. Much of the growing appeal of crime-weather related research in recent times lies in the fact that the world’s climate is rapidly changing (Intergovernmental Panel on Climate Change (IPCC) 2023). Such emergent shifts in climate and associated weather patterns raises concerns regarding the impact that extreme climatic variability will have on various facets of everyday life including health, food security, mobility, and crime (Agnew 2012; Romanello et al. 2023). Regarding the latter, several studies have examined the impact that climate change per se has on crime (see Mares and Moffett 2019; Dadgar et al. 2022; Lynch et al. 2022), with climate change operationalised in a number of ways ranging from long-term climatic changes (Rotton and Cohn 2003; McDowall et al. 2012) to extreme weather events (Mares 2013; Peng and Zhan 2022), and/or temperature anomalies (Schutte and Breetzke 2018; Thomas and Wolff 2023), among others. Regardless, the results of the vast majority of this work have shown how weather variability and volatility are positively associated with the incidence of certain types of crime, especially violent crime (see Schutte and Breetzke 2018; Mares and Moffett 2019). A notable shortcoming of current weather-crime studies, however, is that they have almost exclusively been undertaken within a single city or country. Reasons for this are manifold but could be related to the complexity of standardising crime definitions and reporting practices across different countries, which can vary substantially. Moreover, weather patterns and their impacts can differ widely due to geographic and climatic differences, making it difficult to draw consistent conclusions across locations. Finally, sociocultural factors influencing crime rates vary across countries, adding another layer of complexity to isolating the effects of weather on crime.

In this study we take on this challenge and examine the impact of temperature and rainfall anomalies on violent crime in two comparable coastal mid-latitude locations: Khayelitsha (in South Africa) and Ipswich (in Australia). More specifically, we aim to determine whether there are meaningful associations between violence and anomalies in daily temperature and rainfall. Examining linkages across two different cultural, and situational contexts that exist within similar climatic contexts permits more definitive insights of weather anomalies on violence than has been possible through existing scholarship. Such insights hold important implications for future crime prevention and mitigation strategies in an era of climate change. More broadly, the findings from this study are essential for safeguarding human well-being, promoting social stability, and informing policies and strategies aimed at addressing the complex challenges posed by a changing climate.

## Literature review

A vast number of studies have found how certain types of weather patterns promote violence (see Rotton and Cohn 2003; McDowall et al. 2012; Mares 2013; Xiaofeng et al. 2017; Stevens et al. 2019). With few exceptions (see Ekwall and Lantz 2015; Lynch et al. 2022), the general consensus among scholars is that an increase in temperature in particular is associated with an increase in crime (see Cohn and Rotton 1997; Larrick et al. 2011; Ranson 2014). It is important to note, however, that an increasing number of studies have found a critical threshold beyond which crime does not linearly increase with an increase in temperature but rather plateaus or can even decrease (see Baron and Bell 1976; Gamble and Hess 2012; Schinasi and Hambra 2017; Stevens et al. 2019, 2024a) This so-called curvilinear effect refers to the fact that while moderately high temperatures can cause negative effects (such as increased aggression) and an increase in crime, extremely high temperatures can result in individuals more likely attempting to escape the high temperatures resulting in a reduced crime risk.

A number of factors have also been found to mediate the relationship between temperature in particular and crime. Socioeconomic conditions, such as poverty and unemployment, can exacerbate the impact of high temperatures on crime, as more deprived neighbourhoods may lack the necessary resources to mitigate heat-related stress (see Breetzke and Cohn 2012). Alcohol consumption can also mediate this relationship since alcohol use and abuse has been found to increase in warmer weather (Hagström et al. 2019) and is a well-known risk factor for violence (Mayer et al. 2023). In fact, recent work by Cohen and Gonzalez (2024) found that shifts in alcohol consumption accounted for up to 28% of temperature-induced crimes in Mexico. Finally, police presence and law enforcement practices can vary depending on weather conditions which may also affect crime rates, making it difficult to isolate the direct impact of temperature (Braga et al. 2014). Recent work by Stevens et al. (2024a) has also even found how the location of violence impacts the temperature–aggression association. The researchers found how domestic violence increased with an increase in temperature but that this association was much greater for locations inside compared to outside locations.

Studies have also found that the degree of association between temperature and crime fluctuates based on a range of factors including the type of crime, the temporal and spatial scale, as well as the geographic locale under investigation. For example, an increase in temperature has most often been found to be positively associated with violent crimes such as assault (Breetzke and Cohn 2012; Corcoran and Zahnow 2021) and homicide (McDowall and Curtis 2015), but not necessarily with property crimes (Cohn 1990; Ding and Zhai 2021; Lynch et al. 2022). The notion here – aligned with routine activities (RA) theory - is that an increase in temperatures increases opportunities for socialisation increasing the opportunities for various types of interpersonal crimes. Temporally, studies have examined the crime-temperature linkage at a range of resolutions including annually (Rotton and Cohn 2003; Szkola et al. 2021), monthly (Mares 2013; Churchill et al. 2023), weekly (Ceccato and Uittenbogaard 2014; Jung et al. 2020), daily (Schinasi and Hamra 2017; Schutte and Breetzke 2018) and hourly (de Melo et al. 2018; Towers et al. 2018) with no discernible gradient in strength of positive association across scale. An increasing number of studies have begun to examine the temporally lagged effect of temperature on crime (see Xu et al. 2020; Ankel-Peters et al. 2023). For example, Tiihonen et al. (2017) found a one-month lag effect for high temperatures on violent crime in Finland while Potgieter et al. (2022) found a seven-day lag effect for high temperatures on the relative risk of violent crime in South Africa. The causal pathways linking weather to an increase in crime over time are myriad and are thought to be associated with how weather shocks can disrupt people’s daily routines, leading to increased stress and frustration and increased crime risk (in the short term); and to increase tangential crime risks such as increased inequality (Kelly 2000; Manea et al. 2023), and/or lower agriculture productivity (Somanathan et al. 2021), among others (in the long term).

In terms of spatial scale, the vast majority of research has found positive associations between temperature and violent crime regardless of the spatial scale under investigation. By far the most common spatial scale has been citywide (see Gamble and Hess 2012; Reeping and Hemenway 2020; Xu et al. 2020), followed by district (Berman et al. 2020; Wu et al. 2020) although an increasing number of studies have investigated this association at the neighbourhood-level and below (see Jung et al. 2020; Gorislavsky and Mares 2022). From a location perspective, there has been a tendency for the weather-crime research to focus on Western contexts that experience temperate climates (Corcoran and Zahnow 2022), although studies are now emerging from lesser studied regions of the world including Saudi Arabia (Algahtany et al. 2022), India (Blakeslee and Fishman 2018), Columbia (Trujillo and Howley 2021), and Nigeria (Afon and Badiora 2018), among numerous others. Regardless of the spatial scale or geographic locale, the overall picture that has emerged from this growing body of literature is that higher temperatures increase the probability of interpersonal violence, and criminal behaviour.

Methodologically, researchers have typically applied cross-sectional study designs (Rotton and Cohn 2003; Ranson 2014; Reepingg and Hemenway 2020), and/or used longitudinal data and employed time-series designs to examine the impact of weather on crime (Schinasi and Hamra 2017; Hu et al. 2024). In the majority of these studies a mean (daily, weekly, monthly, annual) temperature is calculated and compared with those during control periods in the same location under study. Other studies have examined the impact that anomalous and/or disproportionate variations in temperatures (or temperature ‘shocks’) have on crime rates (see Jacob et al. 2007; Gangopadhyay and Nilakantan 2018). The notion here is that while broader weather fluctuations may, over time, increase crime risk it is rather extreme and intense spikes in unseasonal weather that may have an acute impact of crime. The rise in the occurrence and destructive power of extreme weather events such as extreme heat or rain is one notable by-product of climate change that is likely to impact various forms of behaviour, including criminal behaviour. That is, extremely hot temperatures will likely increase the discomfort and frustration levels of individuals leading to more aggressive behaviour and ultimately more violent crime. This causal mechanism is substantiated theoretically by the heat-aggression mechanism (Anderson 1989), which argues that hot temperatures increase aggressive motivation and (under certain exigent conditions), aggressive behaviour.

Definitions of what constitutes an ‘anomalous’ weather period vary, however, with most researchers employing a ‘departure-from-historic-normal’ approach to delineate a weather extreme or volatility. For example, Mares et al. (2013) deducted the long-term mean temperature monthly value (from 1971 to 2000) from the actual mean monthly value and extracted anomaly values whereas Churchill et al. (2023) calculated temperature shocks as the difference between the observed temperature and the long-run mean divided by the long-run standard deviation. Other studies have extracted and/or combined the hottest days per year over a five-year study period and compared crime on these days with ‘normal’ or ‘random’ days (see Schutte et al. 2021) or compared daily temperatures departing from more *recent* temperatures to capture an element of unexpectedness and measure weather volatility (see Thomas and Wolff 2023). Regardless, in the vast majority of instances the positive association between temperature and crime holds true.

Studies examining the impact that rainfall specifically has on crime have generally been less forthcoming but studies that have been undertaken have most often produced mixed results with some finding an increase in crime during periods of increased rainfall (see Hsiang et al. 2013; Shen et al. 2020) and others finding no such association (see McLean 2007; Schutte and Breetzke 2018). The operationalisation of rainfall in studies is usually more straightforward and has been measured as the presence or absence of rainfall on any given day, week or month and compared rates of crime on comparable days in which no rainfall is recorded (see Mares 2013; Linning et al. 2017; Sommer et al. 2018).

Despite a lot being known about the weather-crime relationship there are still some notable gaps in the extant literature. As previously mentioned, most previous studies examining this relationship have been undertaken across single cities (see Jung et al. 2020; Reeping and Hemenway 2020; Shen et al. 2020), or across a large number of cities within the same country (see Berman et al. 2020; Xu et al. 2020; Peng and Zhan 2022; Thomas and Wolff 2023) with much fewer studies examining this association across cities in different countries but within the same coastal mid-latitude. The small number of global empirical studies that have been undertaken have largely corroborated the weather-crime linkage, but the results are mixed and nuanced. For example, Rotton (1986) found positive correlations between homicide and a number of climatic variables across 41 countries but found that these positive associations disappeared when a social variable (life expectancy) was partialled out of the analysis. Similarly, Lynch et al. (2020) found that the long-term positive correlation between temperature and homicide in New York and London (for 1895–2015) disappeared when gross domestic product was controlled. Other studies have found weather to have an indirect effect on homicide, mediated by other factors. For example, Barlett et al. (2020) connected global warming to extreme weather events that threaten clean water supplies, which, they inferred, creates resource stresses that motivate criminal behaviour. Finally, Kuznar and Day (2021) examined the causal connections between homicide, inequality, and temperature in 173 countries and found considerable variability in the extent to which these three phenomena combine. Specifically, they found homicide rates to be higher when poorer segments of populations were disproportionately influenced by temperature. Other cross-national studies include Mares and Moffett (2016) who found that each degree Celsius increase in annual temperatures in a sample of 57 countries was associated with a nearly 6% average increase in homicides and Wei et al. (2022) who found a direct and positive relationship between higher temperatures and homicide for 171 countries from 2000 to 2018. The results of these collective cross-national works suggest that examining the weather-crime linkage across contexts is not straightforward but rather is complicated by mediating factors unique to each locale.

In this study we add to what is known in this scholarly space by examining the impact that anomalous temperature and rainfall days have on violent crime in two diverse cities on two separate continents but that are situated in comparable coastal mid-latitude locations. We hypothesise that anomalous weather will be associated with significant shifts in violent crime regardless of context. Research on the relationship between climate and crime is essential for developing effective crime prevention strategies now and in the future. Understanding this connection can help policymakers and law enforcement anticipate and mitigate potential impacts, ensuring safer and more resilient communities.

## Study sites

### Khayelitsha

The township^1^ of Khayelitsha is located approximately 30 km to the south-east of the Cape Town central business district (CBD) in a region colloquially known as the ‘Cape Flats’. The Cape Flats has a unique history in the country. During apartheid, the national government forcibly relocated all non-White communities to the Cape Flats, leading to the creation of so-called ‘townships’ which were (and still are) characterised by high levels of poverty, overcrowding, and inadequate service delivery and infrastructure. Paradoxically, the Cape Flats became a centre of resistance against apartheid, witnessing significant political activism and struggle. Since democracy in 1994, all townships within the Cape Flats have continued to face socio-economic challenges, including rising poverty, high unemployment, and crime, particularly gang violence (see Pinnock 2016). Khayelitsha is among the poorest townships on the Cape Flats with roughly 40% of residents unemployed, and youth unemployment (aged 15–23) at over 50% (Statistics South Africa (SSA) 2011).^2^ The township has a population of roughly 400,000 inhabitants (SSA 2011). Crime is rampant in Khayelitsha with the main policing precinct in the township consistently among the most violent in the country with contact crime^3^ almost double the national average (Crime Hub 2023). In terms of climate, Khayelitsha experiences a moderate climate with mild, wet winters and dry hot summers. The summer season (December to February) brings warmer temperatures, ranging from around 15°C to 28°C, with little to no rainfall and occasional hot days. During the winter months (June to August), temperatures range from around 8°C to 18°C, with occasional rainfall and cool evenings.

### Ipswich

Ipswich is a city located approximately 40 km west of the Brisbane CBD in Queensland, Australia. The city has a diverse population of roughly 250,000 inhabitants (Australian Bureau of Statistics 2022) the majority of whom have an Anglo-European background. Recent decades have, however, seen significant demographic shifts, with increasing cultural diversity due to immigration from countries such as Vietnam, India, and the Philippines. The population of Ipswich is relatively young, with a significant proportion under the age of 30, and the city is known for its affordable housing compared to neighbouring urban centers like Brisbane. According to the Queensland Police Service (QPS 2024), the crime rate in Ipswich is considerably higher than the average across most suburbs in Australia. In fact, Ipswich is ranked in the top 2% of the neighbourhoods across Australia in terms of crime risk. The city typically experiences a subtropical climate characterised by hot, humid summers and mild, dry winters. During the summer months (December to February), temperatures can often soar above 30 °C, occasionally reaching into the mid-40s Celsius during heatwaves.

## Data and methods

### Weather data

Daily weather observations for Khayelitsha and Ipswich were obtained from Open-Meteo (https://open-meteo.com/), an open-source weather API which includes data on a range of meteorological parameters. For each study site, a single location, central to each study area was used based on the underlying latitude and longitude. The weather data obtained for both locations included the apparent daily temperature and rainfall for each of the 1,825 days included in the study period (2011–2015). Apparent temperature is a measure of the ‘feels like’ temperature that uses temperature, humidity, and wind to compute a measure reflecting the experienced temperature (see Yin et al. 2023). This measure was used in the current study given that it offers a more human-centric measure of experienced temperature than other measures (Steadman 1984).

### Crime data

**Fig. 1:**
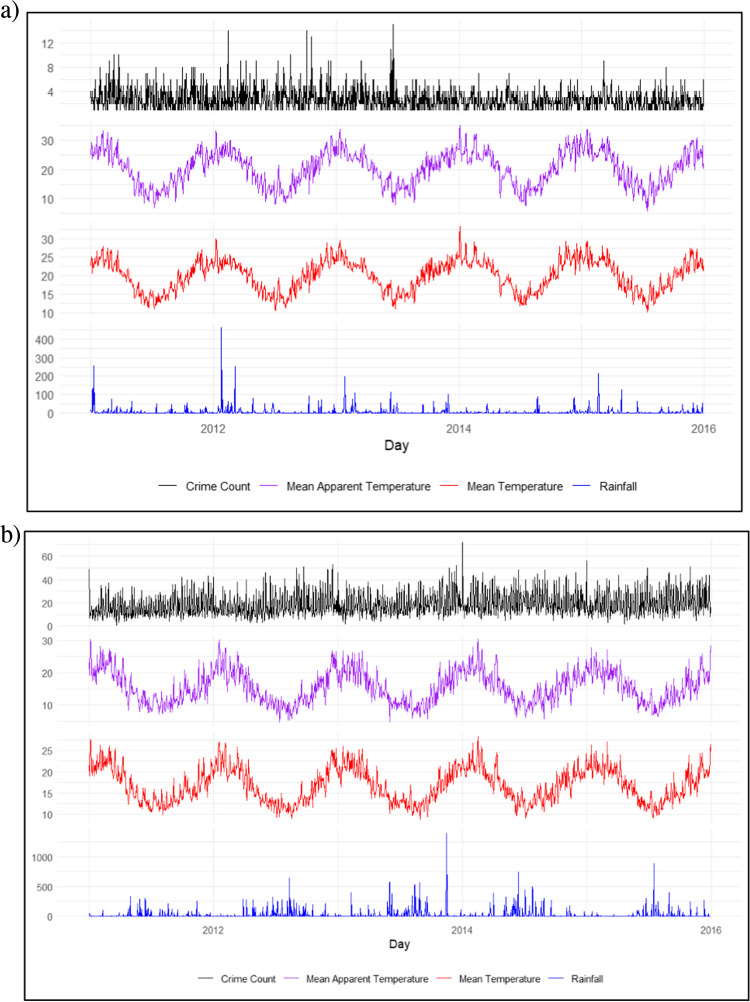
Weather and crime over the study period in: (**a**) Ipswich (Australia); (**b**) Khayelitsha (South Africa). Crime (daily count); Mean apparent temperature (degrees Celsius); Mean temperature (degrees Celsius); Precipitation (daily total rainfall in mm)

Crime data for Khayelitsha was obtained from the South African Police Service (SAPS) for the Khayelitsha police precinct for the same five-year period. Data provided by SAPS included the date, time, and category of each crime incident over the study period. Violent crime incidences (i.e., homicide, assault and robbery) were extracted from the crime incident data resulting in a total of 33,761 crimes (mean per day = 18.5; SD per day = 9.4) over the study period. Crime data for Ipswich were obtained from the Queensland Police Service (QPS) via their online crime mapping service (QPS 2024). A total of 4,121 violent crimes (aligning with the Khayelitsha crime incident data: homicide, assault and robbery) were reported in Ipswich over the study period (mean per day = 2.6; SD per day = 1.7), substantially lower than Khayelitsha. All violent crimes committed in each of our two locations were then used in the empirical analyses. We readily acknowledge that we examine violence as an aggregate category in this study and are aware of the potential impact such aggregations can have on the results (see Andresen and Linning 2012) but we were keen on improving the statistical power of our analysis through aggregation. Considering how weather anomalies might act to re-shape violent crime risk, it is likely that there may be different weather-violence mechanisms at play here - such that homicide, rape, and assault each are impacted in different ways. Future studies should seek to unveil these differences. Table 1 shows the summary statistics for the crime and weather variables employed in the study while Fig. 1 shows the weather and crime variations of the two study locations across the analysis period.

**Table 1:** Summary statistics for the crime and weather variables

	Khayelitsha				Ipswich			
	Min	Max	Mean	SD	Min	Max	Mean	SD
Crime (count)	1	72	18.5	9.36	1	15	2.67	1.75
Mean apparent temperature (°C)	4.80	30.30	15.51	4.96	5.90	35.00	20.57	5.77
Mean temperature (°C)	8.90	28.30	16.80	3.97	10.20	33.30	20.08	4.15
Precipitation (mm)	0	38.80	1.31	4.12	0	199.5	2.03	7.98

### Analysis

Initially, we analyse simple counts of violent crime by apparent temperature—using three groups: low, medium, and high— and rainfall using two categories: dry and wet—for visual comparison. A ‘low’ apparent temperature is more than a standard deviation below the mean apparent temperature over the full period, and a ‘high’ apparent temperature is more than a standard deviation above. We then detect days subject to a weather anomaly. A daily weather anomaly is operationalised as statistically meaningful departures from recent weather conditions. These departures include both unseasonably warm or unseasonably cold temperatures, and/or rainfall events. Weather anomalies are measured as daily temperatures or rainfall events that are at least two standard deviations higher or lower than the 30-day rolling daily average.^4^ Visualisations for each case study site (temperature anomalies per standard deviational difference) were calculated and a composite graphic displaying standard deviational increases (on the x-axis) and the daily crime rate (on the y-axis), relative to the baseline crime rate was determined. We examine these anomalies in aggregate across a 12-month period as well as seasonally for both summer and winter. Next, we analyse the potential confounding effect of weekends on the weather-crime relationship across these two contexts. That is, we detected days subject to a weather anomaly (temperature and rainfall) on weekends versus weekdays. Weekends were defined as the period between Saturday at 1200am local time and Sunday at 2359pm local time. Conversely, weekdays were defined as the period outside this time frame. Again, visualisations for each case study site were calculated and a composite graphic displaying standard deviational increases and the daily crime rate, relative to the baseline crime rate was determined.

Finally, we examine the relationship between anomalous weather and violence in socio-economically disadvantaged neighbourhoods in both locations. Recent evidence suggests that the impact of anomalous weather conditions on violence may not be uniformly felt across neighbourhoods stratified by a range of socio-demographic factors, notably disadvantage (see Breetzke and Cohn 2012; Berman et al. 2020; Heilmann et al. 2021). We extend our analysis here to determine whether this is also the case across these two diverse locations. For Khayelitsha, ‘economic disadvantage’ is operationalised by focusing on neighbourhoods where the resident population earn less than R64,000 per annum (US$3465), which equates to roughly 10% of the population. In Ipswich, this is operationalised as neighbourhoods that were classified in the 10% most disadvantaged using data available through the Australian Bureau of Statistics. Specifically, we use the Socio-Economic Indexes for Areas (SEIFA) information that ranks the relative socio-economic advantage and disadvantage of all neighbourhoods across Australia using Census data (Australian Bureau of Statistics 2021). We readily acknowledge that these measures are different and are somewhat of a crude representation of ‘economic disadvantage’ but our intention here is simply to briefly examine whether the relationships we observe across both locations, as a whole, change if we extract a subset of neighbourhoods. One-way ANOVA and Tukey’s tests for multiple pairwise comparisons are used to identify statistical meaningful differences (if any) alongside the use of visualisations capturing the anomalous weather-violence relationship. Ethics approval for the study was granted by the University of Pretoria (reference number: NAS366/2019).

## Results

Table 2 shows the apparent temperature upward and downward anomalies from the 30-day rolling average temperature in Khayelitsha and Ipswich respectively. We include the number of days that an anomaly from the norm was observed as well as the mean of the average rolling temperature on the days an anomaly was observed. Importantly, the 30-day rolling temperature represents the mean of the 30-day rolling average preceding the days on which a temperature or rainfall anomaly was detected. The value does not represent the average of the full period of analysis. Finally, we add the mean and standard deviation of those anomalous days. The 30-day rolling temperatures observed for Khayelitsha for the upward temperature anomaly was over 25% lower than for Ipswich indicating an overall colder climate. For the downward temperature anomaly, the 30-day rolling temperature was also lower in Khayelitsha compared to Ipswich. Using our definition of two standard deviations above the 30-day rolling average in each location, 6.5% of days across the study period were defined by an upward departure in temperature (than usual) in Khayelitsha, compared to 5.9% of days in Ipswich. Percentages were also broadly similar between cities for downward departures from in temperature with 3.2% of days across the study period were defined by downward departure in temperature in Khayelitsha compared with 4.3% of days in Ipswich. In terms of rainfall, approximately 6.2% of days in Khayelitsha over the study period (*n* = 114 days) experienced an upward departure from ‘normal’ rainfall compared with 6.4% of days in Ipswich (*n* = 119) (see Table 3).

**Table 2:** Temperature anomalies for Khayelitsha and Ipswich (2011 to 2015)

Location	Upward temperature anomaly					Downward temperature anomaly				
	Days (*n*)	30-day rolling temperature^a^		Temperature anomaly		Days (*n*)	30-day rolling temperature		Temperature anomaly	
		Mean	SD	Mean	SD		Mean	SD	Mean	SD
Khayelitsha	119 (6.5%)	14.57	0.47	20.15	4.94	58 (3.2%)	16.01	0.60	10.62	3.39
Ipswich	109 (5.9%)	19.56	0.52	24.61	5.57	81(4.3%)	19.30	0.47	13.84	4.01

^a^This value represents the mean of the 30-day rolling average preceding the days on which a temperature or rainfall anomaly was detected. This value does not represent the mean of the full period of analysis.

**Table 3:** Rainfall anomalies for Khayelitsha and Ipswich (2011 to 2015)

Location	Upward rainfall anomaly				
	Days (*n*)	30 day rolling average		Rainfall anomaly	
		Mean	SD	Mean	SD
Khayelitsha	114 (6.2%)	0.99	2.16	12.34	9.60
Ipswich	119 (6.4%)	1.49	3.42	20.74	26.74

**Fig. 2:**
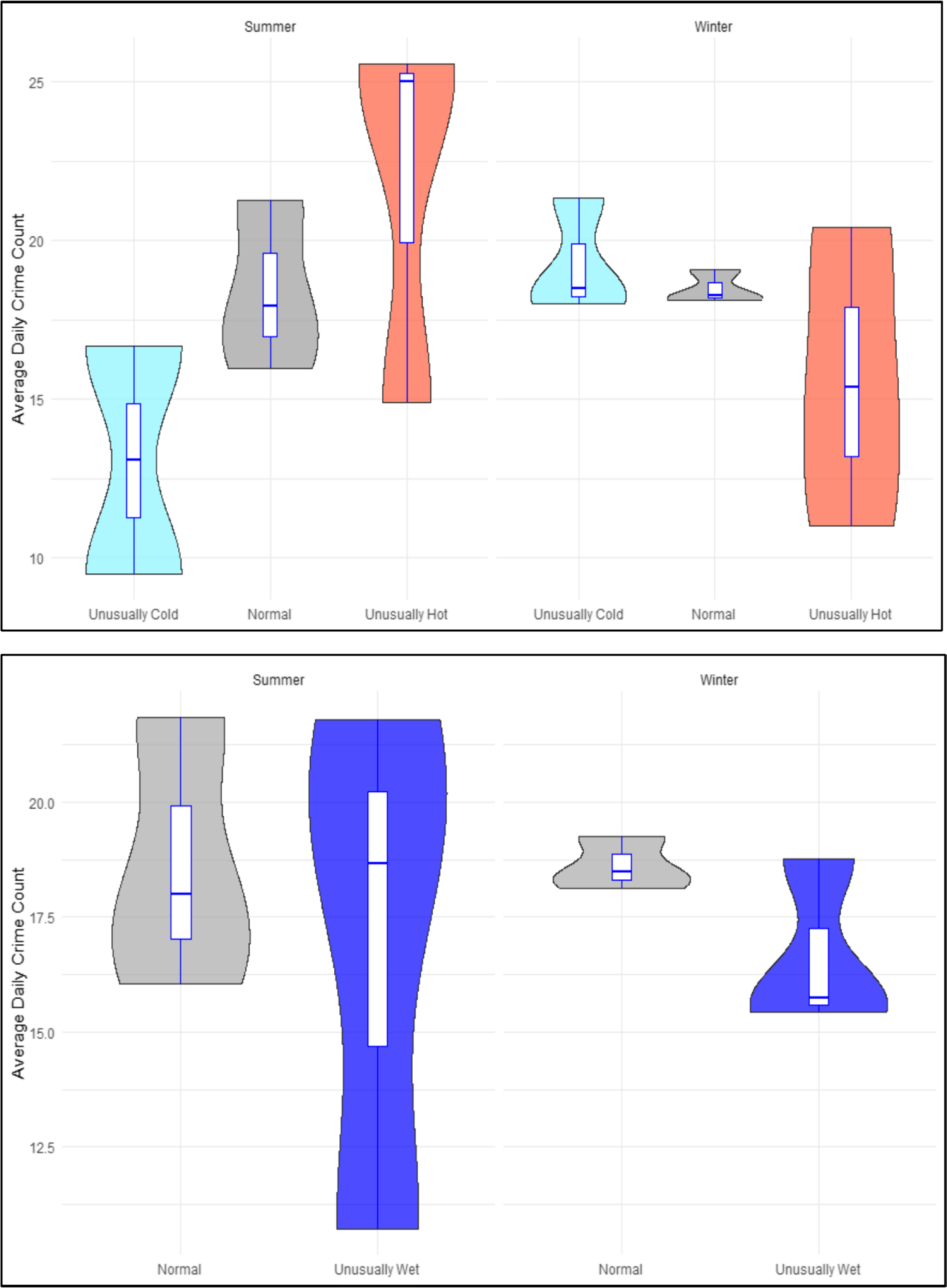
Associations of heat and rain against violent crime in Khayelitsha (South Africa)

**Fig. 3:**
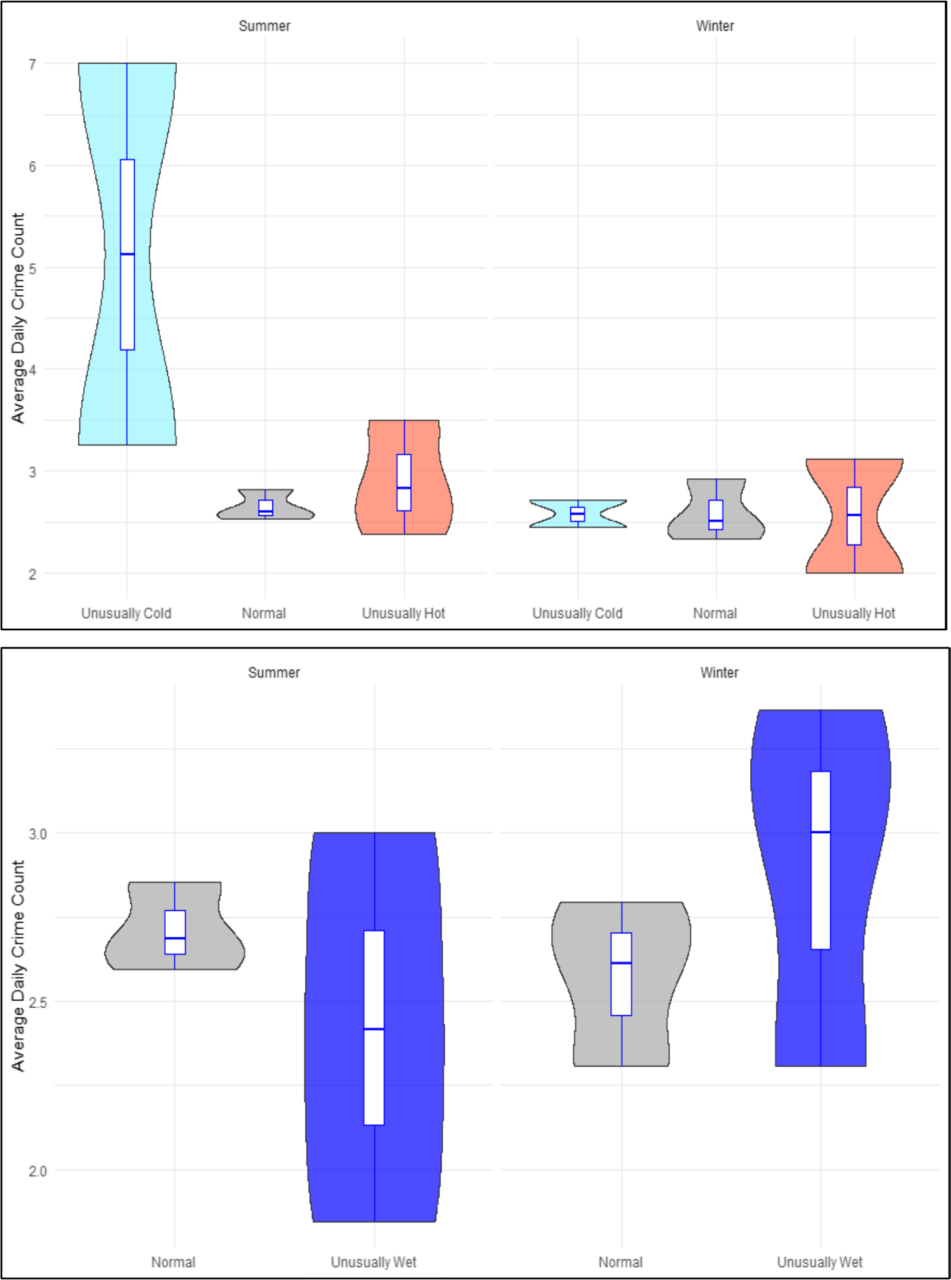
Associations of heat and rain against violent crime in Ipswich (Australia)

Figure 2 provides a comparison of the mean daily violent crime counts in Khayelitsha on days with different meteorological temperature exposures in summer and winter respectively^5^. Mean daily violent crime was found to be the highest in unusually hot days in summer when compared to ‘normal’ and unusually cold days. Results are less variable in winter although the mean daily violent crime was found to be considerably lower on unusually hot days in winter compared to ‘normal’ and unusually cold days. In terms of rainfall, mean daily violent crime in Khayelitsha is marginally higher in unusually wet days in summer compared to ‘normal’ days but is considerably lower in unusually wet days in winter compared to ‘normal’ days. In comparison, mean daily violent crime is found to be the *highest* in unusually cold days in summer in Ipswich compared to ‘normal’ and unusually hot days (see Fig. 3). In terms of rainfall, there appears to be very little impact of different rainfall exposures on violence in Ipswich. Results of the one-way ANOVA found that unusually hot days in Khayelitsha exhibited significantly higher mean daily crime counts compared to ‘normal’ days (*p* < 0.05) over the study period. No significant differences were found in terms of violent crime in Khayelitsha between normal days and unusually wet days (*p* = 0.205). Surprisingly, no significant difference was found between violent crime committed on unusually hot day (*p* = 0.99) or unusually cold days (*p* = 0.89) in Ipswich compared to ‘normal’ days. Moreover, no significant differences were also observed between violent crime on unusually wet rainfall days (*p* = 0.19).

**Fig. 4:**
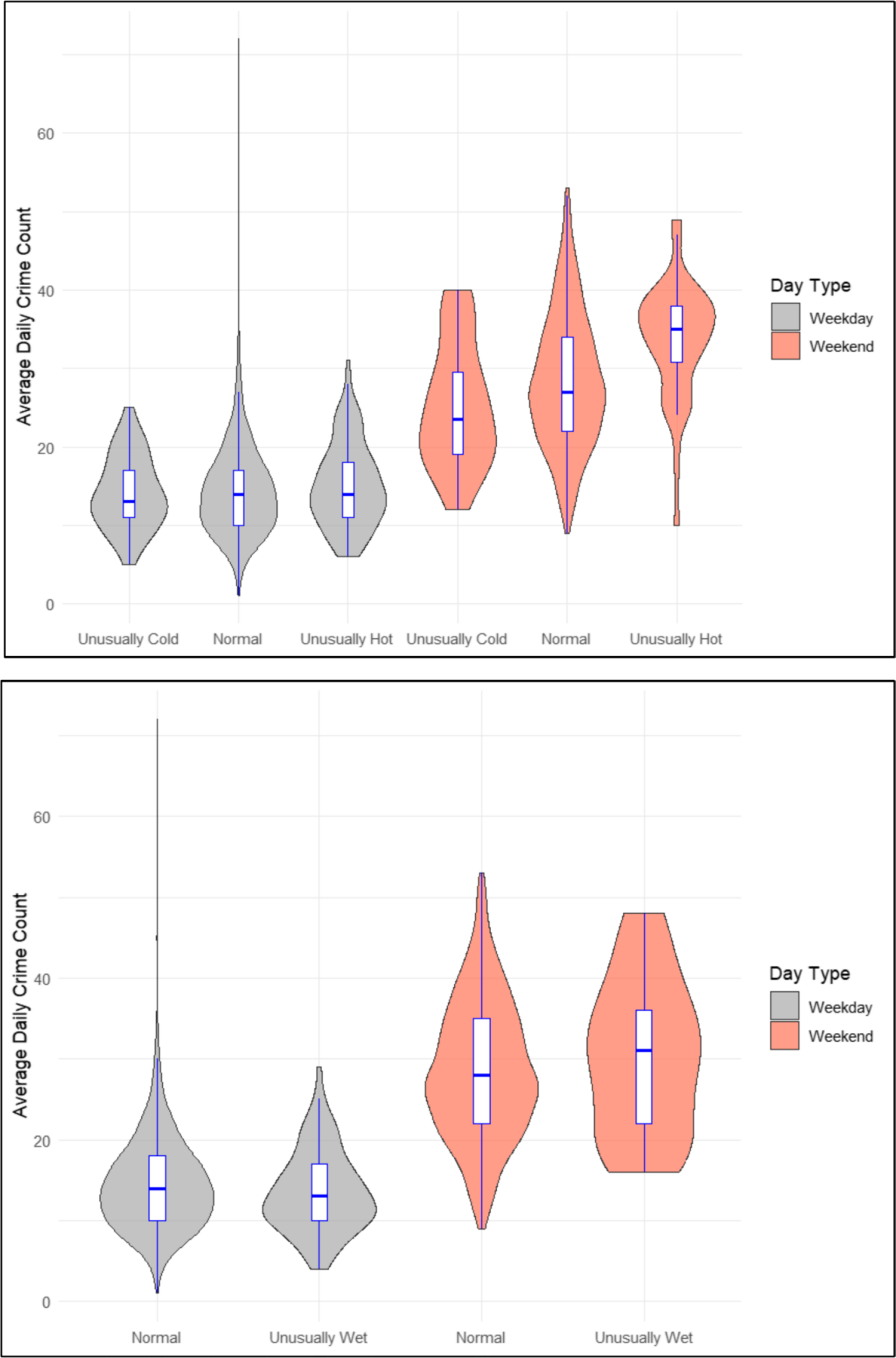
Associations of heat, and rain against violent crime in Khayelitsha (South Africa)

**Fig. 5:**
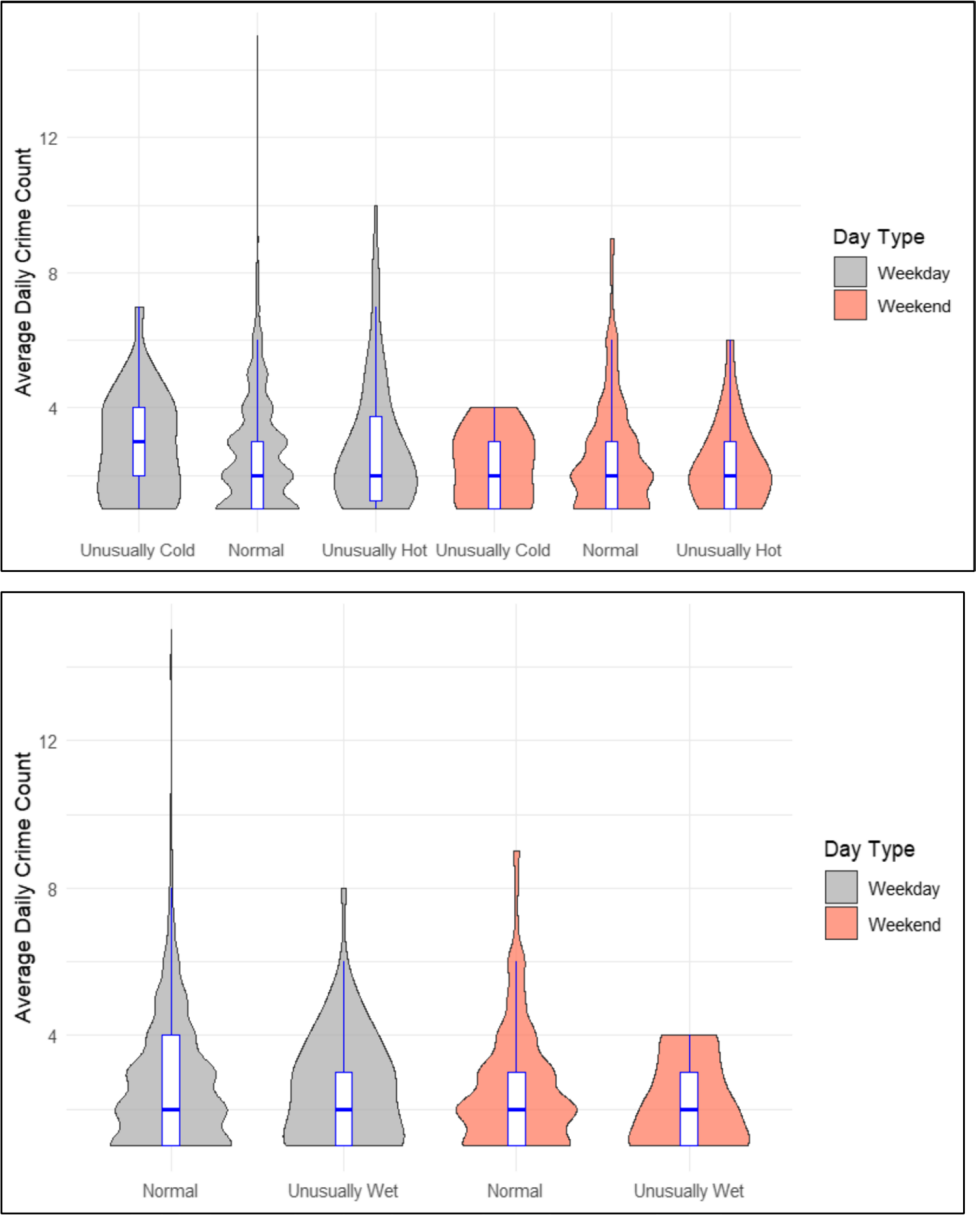
Associations of heat, and rain against violent crime in Ipswich (Australia)

Figure 4 provides a comparison of the mean daily violent crime counts in Khayelitsha on weekend versus weekdays with different meteorological temperature exposures. Mean daily violent crime was found to be uniformly, and significantly, higher on weekends compared to weekdays regardless of the temperate exposures (*p* < 0.001). In terms of temperature anomalies and violence between weekends and weekdays, all categories were found to be significant with violence significantly higher on ‘normal’ weekends compared to ‘normal’ weekdays (*p* < 0.001); on unusually cold weekends compared to unusually cold weekdays (*p* < 0.001); and on unusually hot weekends compared to unusually hot week days (*p* < 0.001). In terms of rainfall anomalies and violence both categories were, again, found to be significant with violence significantly higher on ‘normal’ weekends comparted to ‘normal’ week days (*p* < 0.001); and on unusually wet weekends compared to unusually wet weekdays (*p* < 0.001). In comparison, one-way ANOVA and Tukey’s tests for multiple pairwise comparisons revealed no significant differences in violence between weekends and weekdays, or between ‘normal’ and anomalous weather conditions in Ipswich (see Fig. 5).

Finally, results of the analysis examining the impact of anomalous temperatures and rainfall on violence by economic deprivation were found to be similar to the results above. That is, unusually hot days in Khayelitsha, again, exhibited significantly higher mean daily crime compared to ‘normal’ days, although this difference increased in significance (*p* < 0.01) in the most economically disadvantaged neighbourhoods. No significant differences were found in violent crime in Khayelitsha between normal days and unusually wet days (*p* = 0.08) in the most economically disadvantaged neighbourhoods. Similar to the initial analysis for Ipswich, no significant associations were found between anomalous temperature and rainfall days on violence by economic deprivation in Ipswich at all with no measures approaching significance when analysis was confined to the most economically disadvantaged neighbourhoods.

## Discussion

Cross-national studies examining the weather-crime relationship are relatively uncommon. Most prior research has examined this linkage in a single city and/or country (see Jung et al. 2020; Reeping and Hemenway 2020; Peng and Zhan 2022; Thomas and Wolff 2023), and not considered whether the results obtained are comparable across diverse cultural and social contexts. This is important to ascertain as cross-national comparative studies such as this offer a much broader perspective on the weather-crime association as they enable researchers to identify generalisable patterns and validate findings across diverse datasets. In contrast, in this study we examine the impact of anomalous temperature and rainfall days on crime across two different settings. Importantly, both locations are located at the coast and are within the same hemisphere, and same broad latitude. The results of our analysis indicate some notable differences in the impact of temperature and rainfall anomalies on violent crime in these two locations. For Khayelitsha, anomalously hot days were found to exhibit significantly higher rates of violent crime than other days while no such association was found for Ipswich. In terms of rainfall, no significant associations were found in either context. A stratification of the data across both contexts by weekend and weekday found that violence was significantly affected by this confounder in Khayelitsha in particular. That is, violence was significantly higher in this context on weekends regardless of the temperate and rainfall exposures. Importantly, however, mean daily violent crime was still found to be the *highest* in unusually hot weekend days compared to ‘normal’ and unusually cold weekend days in this context. A final stratification of the data across both contexts by economic disadvantage found that the most disadvantaged neighbourhoods in Khayelitsha experienced an even stronger relationship between mean daily violence and anomalously hot days while no significance was, again, observed in Ipswich. For rainfall, results were non-significant for both contexts in neighbourhoods stratified by economic disadvantage.

For Khayelitsha, our results are broadly similar with the vast number of studies that have examined the temperature-crime linkage both globally (Mares and Moffett 2016; Kuznar and Day 2021) and locally (Breetzke and Cohn 2012; Potgieter et al. 2022). That is, an increase in temperature increases crime risk although this was only found to be the case in summer. Our study however focussed specifically on the impact of anomalously hot or cold days on violence with results indicating significant differences in crime for Khayelitsha but not for Ipswich. Reasons for these contrasting results are speculative but could be found in the underlying socio-demographics of both locations. In terms of the former, Khayelitsha is extremely poor. According to the World Economic Forum (2016) the township is one of the world’s five biggest slums with an estimated 32–46% of households living in extreme poverty. Residents in impoverished settings such as Khayelitsha may be less able to escape the heat (on unusually hot days) than residents in more affluent settings such as Ipswich. Importantly, this is not a new nor novel insight. Over thirty years ago Harries and Stadler (1983) postulated that the stronger relationship found between temperature and assault in low socio-economic neighbourhoods in Dallas, Texas, was due to the fact that the more affluent could potentially escape hotter temperatures. Later, Harries et al. (1984) showed that the hot summer effects on aggressive crime rates in Dallas were most pronounced in neighbourhoods where air conditioning was scarce. Twenty years later, Rotton and Cohn (2004) found that assaults in climate-controlled settings remained fairly constant over time, but that there was a linear increase in crimes in areas that probably lacked climate control while in São Paulo, Brazil Ceccato (2005) found seasonal homicide occurred more frequently in summer and winter in the disadvantaged areas in the south of the city. More recently, Heilmann et al. (2021) found the heat-crime relationship to be much stronger in neighbourhoods in Los Angeles with higher levels of poverty. More specifically, they found that neighbourhoods with a 10% increase in the share of families living under the poverty line experienced more crime from a 1.8-degree Celsius increase in daily maximum temperature than other neighbourhoods. The researchers ascribe these differences, in part, to differences in non-cognitive (social-emotional or personality) skills between residents in high versus low income households. In our study we similarly found that the relationship between anomalously hot days on violence was strongest in the poorest neighbourhoods, at least in Khayelitsha, which provides further evidence for disadvantage being an important mediating factor in this relationship, in some contexts.

Another possible reason for the contrasting results we found in our study could be related to the differing natural environments in each location. More specifically, the ability of certain aspects of the natural environment such as urban greenery to not only reduce the effects of urban heat islands (and thereby reduce ambient temperature) but to create a recreational outlet for residents to escape the heat (see Stevens et al. 2024b). Ipswich has over 550 urban parks and reserve areas covering in excess of 8,500 hectares (Ipswich City Council (ICC) 2024), with roughly 45% of the city having a greenspace covering (ICC 2024). In contrast, Khayelitsha was created in the early 1980s as a result of the forced removal of non-White residents from central Cape Town into outlying areas distant and distinct from the former White urban core. The township is notoriously under-developed with relatively few formal built facilities. In fact, the first shopping centre was only constructed in 2005 to serve a population of 400,000. In terms of urban greenery, Khayelitsha has roughly 53 community and district parks; this for an area similar in population size to Atlanta (in the US) or Liverpool (in the UK). The informal areas of Khayelitsha – which comprises roughly 40% of the township - have no parks or playgrounds whatsoever with the density of the settlement being so high that there are no clear and clean spaces for residents to socialise close to their houses (Smit et al. 2016). Moreover, the urban greenspaces that are available are badly maintained (Mathenjwa 2017) and considered dangerous by residents (Smit et al. 2016). The main urban park in the township - the Khayelitsha Wetlands Park - is plagued with poor water quality linked to a number of anthropogenic influences including the discharge of treated and untreated waste from both local agricultural and urban waste sources (Mathenjwa 2017). Recent work by Stevens et al. (2024a) has found how the temperature-related risk of violence is increased by being inside. If residents of Khayelitsha do not have safe recreational outlets available to escape the heat and are confined indoors, this could potentially increase the risk of violence. The fact that the results of our research indicate that the poorest of the poor in Khayelitsha (the most economically disadvantaged) suffer the most from an unusually hot day suggests that this divergence in results between these two locations is most likely due to their considerable socio-economic and, and potentially environmental differences too.

Our findings also indicate the significant impact that weekends exhibit on violence in Khayelitsha in particular. Indeed, mean daily violent crime was found to be significantly higher on weekends compared to weekdays in this location regardless of the temperate exposures while no discernible impact was found for Ipswich. Previous studies have most often found an increase in crime on weekends compared to weekdays both locally (Breetzke 2015) and internationally (Andresen and Malleson 2015; Butke and Sheridan 2010). Of course, weekends can be considered as a proxy for socialisation, and/or the increased consumption of alcohol with individuals most often having more free time on weekends. The consumption of alcohol in Khayelitsha is high with more than half of all patients reporting violence-related injuries in the township being under the influence of alcohol at the time of violent incident (Matzopolous et al. 2017). According to the Western Cape Liquor Licensing Authority (2024) there are exactly 140 outlets with valid liquor licenses in Khayelitsha but a study by Matzopolous and colleagues (2017) mapped the location of over 1400 outlets in the township which shows the size of the illicit trade in alcohol in this location. Individuals residing in more deprived neighbourhoods are more likely to experience problematic drinking patterns, including binge drinking and alcoholism (Cerdá et al. 2010; Fone et al. 2013) which could potentially explain why weekends exhibited a much greater impact on mean daily violent crime rates in Khayelitsha and not Ipswich although more research is required to validate this assertion.

Finally, it is worth noting that there are various other potential mechanisms that may explain the impacts of temperature and rainfall deviations from typical levels on violence in these contexts that we have not investigated here. Among these are the presence and visibility of law enforcement which can potentially be affected by weather conditions, as can the frequency and nature of community activities and alternative interventions aimed at preventing crime such as neighbourhood watch programmes. These are particularly valid in the context of Khayelitsha which has experienced a complete breakdown in relations between the community and the police over the past decade (O’Regan et al. 2014). Investigating these was, however, simply beyond the scope of this empirical work, but future analysis could aim to use additional analytical techniques (see Le and Nguyen 2024) to identify other possible mediating factors.

## Conclusion

In their systematic review of the extant weather-crime literature, Corcoran and Zahnow (2021) found a total of 200 studies published between 1842 and 2021 examining this linkage. Among their recommendations for future studies, they highlighted a need for greater exploration of the weather-crime relationship in cultural and climatic contexts outside the United States and the United Kingdom. In this study, we took up this challenge and determined whether there were meaningful associations between violence and anomalies in daily temperature and rainfall in two socio-demographically different contexts. We found some similarities in results in this cross-national study – notably the non-significance in rainfall anomalies between both contexts - but also some notable differences which we attribute to the inherent socio-demographic differences between these two locations. By examining variations in crime rates and weather conditions between countries, researchers can develop more robust theories, inform policy decisions, and identify effective crime prevention strategies that are adaptable to various environmental contexts. Additionally, cross-national studies such as ours can help elucidate the complex interplay between weather, socioeconomic factors, and crime, contributing to a more nuanced understanding of this phenomenon on a global scale. We believe that we have made a small but meaningful contribution in this regard.

## Supplementary Information

Below is the link to the electronic supplementary material.

ESM 1 (DOCX 14.2 KB)

## Data Availability

The datasets generated during and/or analysed during the current study are available from the corresponding author on reasonable request.

## Footnotes

1 In South Africa, a ‘township’ refers to a built-up residential area on the periphery of former Whites-only urban neighbourhoods. Under the spatial planning policies of the former apartheid government, these areas were reserved for non-White people only (i.e., Black African, Coloured and Indian population groups) although townships are still predominantly (> 95,0%) inhabited by non-Whites.

2 This was the most recent census undertaken for which data is available. The 2021 census was postponed due to the Covid-19 pandemic and was undertaken in July 2022. The results of this census have yet to be fully disseminated.

3 ‘Contact crimes’ refer to when a person or people are injured/harmed or threatened with injury/harm during the commission of a crime. A further sub-category of ‘contact-related crime’ is used for violent crimes committed against property with the intention of causing damage to a person, for example arson or malicious damage to property.

4 This approach to detecting days that are subject to anomalous weather patterns follows that employed by Thomas and Wolff (2023).

## Code Availability

Available on request.
