# Supplementary material for: The impact of weather anomalies on violence in the coastal mid-latitudes: a cross-national comparison
Source: Int J Biometeorol. 2024 Sep 6;69(10):2425–40. doi: 10.1007/s00484-024-02762-x (PMC12540588; doi:10.1007/s00484-024-02762-x)
Supplement: Supplementary file 1 — (DOCX 14.2 KB) [file 484_2024_2762_MOESM1_ESM.docx]

**The impact of weather anomalies on violence in the coastal mid-latitudes: A cross-national comparison**

Gregory Breetzke^1*^ and Jonathan Corcoran^2^

^1^ Department of Geography, Geoinformatics and Meteorology, University of Pretoria, Pretoria, South Africa

^2^ School of the Environment, Faculty of Science, University of Queensland, Brisbane, Australia

* Corresponding author: Prof Gregory Breetzke (greg.breetzke@up.ac.za)

**ORCIDs**

Gregory Breetzke 0000-0002-0324-2254

Jonathan Corcoran [0000-0003-3565-6061](https://orcid.org/0000-0003-3565-6061)

**The impact of weather anomalies on violence in the coastal mid-latitudes: A cross-national comparison**

**Abstract**

Cross-national studies examining the relationship between weather and crime are rare. Reasons are manifold but include the differences in countries due to geographical, climatic, and seasonal variations. **In contrast in this study we examine the causal impact of temperature and rainfall anomalies on violent crime in locations located in two comparable geographic zones: Khayelitsha (in South Africa) and Ipswich (in Australia).** We use ANOVA and Tukey's tests to identify statistical meaningful differences (if any) in the impact of these weather anomalies on crime alongside the use of visualisations capturing the anomalous weather-violence relationship in these two contexts. Results show some similarities but also notable differences between locations which we attribute to their inherent socio-demographic differences which we expand upon. We conclude by highlighting the benefits of cross-national crime research, and motivate for its increased application in future research of this nature.

**Keywords:** violent crime, temperature, rainfall, Khayelitsha, Ipswich

**Declarations**

**Funding**: Not applicable

**Conflicts of interest/Competing interests**: The authors have no conflicts of interest to declare that are relevant to the content of this article

**Availability of data and material**: The datasets generated during and/or analysed during the current study are available from the corresponding author on reasonable request.

**Code availability**: Available on request

**Ethics approval**: Details in manuscript

**Consent to participate**: Not applicable

**Consent for publication**: Not applicable

**Acknowledgements:** The authors would like to thank Francois Schutte for compiling the baseline dataset used in this study.
